# Genome-wide identification of the KNOTTED HOMEOBOX gene family and their involvement in stalk development in flowering Chinese cabbage

**DOI:** 10.3389/fpls.2022.1019884

**Published:** 2022-11-10

**Authors:** Xi Ou, Yudan Wang, Jingyi Li, Jiewen Zhang, Zhenbin Xie, Bing He, Zhehao Jiang, Yuting Wang, Wei Su, Shiwei Song, Yanwei Hao, Riyuan Chen

**Affiliations:** College of Horticulture, South China Agricultural University, Guangzhou, China

**Keywords:** flowering Chinese cabbage, knotted1-like Homebox, expression analysis, hormone response, cold response

## Abstract

Gibberellin and cytokinin synergistically regulate the stalk development in flowering Chinese cabbage. KNOX proteins were reported to function as important regulators of the shoot apex to promote meristem activity by synchronously inducing CTK and suppressing GA biosynthesis, while their regulatory mechanism in the bolting and flowering is unknown. In this study, 9 *BcKNOX* genes were identified and mapped unevenly on 6 out of 10 flowering Chinese cabbage chromosomes. The *BcKNOXs* were divided into three subfamilies on the basis of sequences and gene structure. The proteins contain four conserved domains except for BcKNATM. Three BcKNOX TFs (BcKNOX1, BcKNOX3, and BcKNOX5) displayed high transcription levels on tested tissues at various stages. The major part of *BcKNOX* genes showed preferential expression patterns in response to low-temperature, zeatin (ZT), and GA_3_ treatment, indicating that they were involved in bud differentiation and bolting. *BcKNOX1* and *BcKNOX5* showed high correlation level with gibberellins synthetase, and CTK metabolic genes. *BcKONX1* also showed high correlation coefficients within *BcRGA1* and *BcRGL1* which are negative regulators of GA signaling. In addition, BcKNOX1 interacted with BcRGA1 and BcRGL1, as confirmed by yeast two-hybrid (Y2H) and biomolecular fluorescence complementation assay (BiFC). This analysis has provided useful foundation for the future functional roles’ analysis of flowering Chinese cabbage *KNOX* genes

## Introduction

The *KNOX* genes of Arabidopsis thaliana are clustered into three classes, class I, class II, and class M (Magnani and Hake, 2008; Mukherjee et al., 2009). Class I consists of four genes: *SHOO-TMERISTMELESS* (*STM*), *KNAT1* (*BP*), *KNAT2*, and *KNAT6* and specifically expresses in the shoot apical meristem (SAM) (Hake et al., 2004). Four class II *KNOX* (*KNAT3*, *KNAT4*, *KNAT5*, *KNAT7*) genes express more broadly compared with that of class I (Hake et al., 2004). Class I and class II KNOX proteins contain four conserved domains (Reiser et al., 2000; Hamant and Pautot, 2010). The KNOX1 and KNOX2 domains also known as the MEINOX domain are located at the N-terminal portion (Bellaoui et al., 2001; Müller et al., 2001; Smith et al., 2002). The ELK domain functioned as nuclear localization signal is located on the upstream of the homologous domain (Hofer et al., 2001). Moreover, the C-terminal region possess a DNA binding HD domain (Scofield and Murray, 2006). However, class M represents by KNATM which is characterized with missing the ELK and HD domains (Magnani and Hake, 2008).Numerous studies have indicated that class I *KNOXs* play vital roles in meristem maintenance, hormone homeostasis, internode elongation, and inflorescence architecture (Semiarti et al., 2001; Tsuda et al., 2011; Tsuda and Hake, 2015). In maize, loss-of-function mutations of homeobox gene, knotted1, shows shorter meristems (Kerstetter et al., 1997; Vollbrecht et al., 2000). Similar phenotype is observed in rice loss-of-function mutants of *OSH1* (Tsuda et al., 2011). Moreover, another rice homeobox gene *OSH15* plays a dispensable role in the architecture of internodes (Sato et al., 1999). Recent studies have showed that *KNOX* played significant role in abscission. In litchi, *KNAT1* represses fruitlet abscission (Zhao et al., 2020). Meanwhile, the silencing of KNOTTED1-LIKE HOMEOBOX PROTEIN1 (KD1) in tomato also shows a repression of pedicel and petiole abscission (Ma et al., 2015). In model plant Arabidopsis, *KNOX2* and *KNOX6* play a role in carpel development and lateral root formation, respectively (Pautot et al., 2001; Dean et al., 2004). Whereas the functions of the *Arabidopsis* class II *KNOXs* are largely unknown which is in contrary to the well-defined class I *KNOX* genes (Hay and Tsiantis, 2010). In legume, KNOX4 would directly bind to the promoter of a cuticle biosynthetic gene and regulate seed physical dormancy (Chai et al., 2016). Besides that, class II *KNOX* genes involve in legume root nodule development (Di Giacomo et al., 2017). In addition, KNOX7 is a vital regulator of secondary wall biosynthesis in Arabidopsis, rice, and populus (Zhong et al., 2008; Li et al., 2012; Yu, 2019).

Additionally, numerous studies have shown *KNOX* genes affect the metabolism and signaling transduction pathway of hormones, such as ethylene, gibberellin, cytokinin and abscisic acid (Kim et al., 2013). KNAT1 would suppress ethylene biosynthesis by directly binding to the promoters of ethylene biosynthetic genes (Zhao et al., 2020). KD1 of tomato is involved in regulating the genes related to auxin transport and signaling (Ma et al., 2015). The isopentenyl transferase (*IPT*) and cytokinin dehydrogenase (CKX) are key genes in biosynthesis and metabolic of cytokinins (Frébort et al., 2011). Moreover, KNOX proteins activate both CTK biosynthesis and signaling transduction (Kusaba et al., 1998; Ori et al., 1999; Frugis et al., 2001; Jasinski et al., 2005). Class I KNOX proteins were known to promote expression of *IPT7* (Jasinski et al., 2005), while no studies have shown that KONX proteins would regulate the expression of *CKX*. KNOX proteins are also reported to suppress the accumulation of GA (Sakamoto et al., 2001; Chen et al., 2004). Previous studies have shown that overexpression of *POTH1* (potato homeobox 1) results in lower levels of *GA20ox1* (a key rate-limiting enzyme in the synthesis of GA) transcription (Rosin et al., 2003) Moreover, in *Arabidopsis*, *GA3ox1* (GA-biosynthesis gene) transcript shows no difference in KNOX1-overexpression plants (Hay et al., 2002), indicating that *GA20ox1* is a specific target in the GA-biosynthesis pathway for KNOX proteins. Several recent studies have revealed that gibberellin and cytokinin play vital roles throughout bolting and flowering (Guan et al., 2021; Ou et al., 2022). Meanwhile, our preliminary results indicated that GA and cytokinin exert antagonistic effect on bolting in flowering Chinese cabbage (Ou et al., 2022). We may speculate that *BcKNOX* family have significant functions in bolting and flowering of flowering Chinese cabbage yet to be discovered.

Despite extensive studies within some model plants, little is known about the features of *KNOX* genes in flowering Chinese cabbage. In this study, nine *BcKNOXs* were identified and characterized in Brassica campestris genome. The subcellular locations of the majority of BcKNOXs were further analyzed. The expression patterns in various tissues at different stages and in response to cold stress and flowering-related hormones were also explored. The results provide insight into *KNOX* gene family of flowering Chinese cabbage and a foundation for further research on their function.

## Materials and methods

### Identification of *KNOX* genes and phylogenetic analysis

To identify genes encoding KNOX protein in *Brassica campestris* genome, the amino acid sequences of *Arabidopsis thaliana KNOX* genes were used as queries by using BLASP program. Meanwhile, we used the NCBI Conserved Domains database (Marchler-Bauer et al., 2016) and Pfam database (Mistry et al., 2020) to examine genes that have the conserved domains. Protein basic physicochemical characteristics and subcellular location were predicted with ExPASy program (Gasteiger et al., 2003) and WoLF PSORT (Horton et al., 2007) respectively. Phylogenetic tree including KNOXs from *Arabidopsis thaliana*, *Brassica rapa* and *Brassica campestris* was constructed utilizing MEGA11 (Tamura et al., 2021)

### Chromosomal location and multiple sequence alignment of KNOX genes

TBtools software was used to map the Chromosomal position of *BcKNOX* genes. Multiple alignment was conducted by ClustalW based on the full-length amino acid sequences of KNOX proteins from *Arabidopsis* and *Brassica campestris*.

### Synteny analysis

Synteny analysis was carried out using the Toolkits of TBtools software (Chen et al., 2020). MCScanX was applied to identify syntenic blocks and distinct duplication events within Brassica campestris and between *Arabidopsis* and *Brassica campestris*.

### Gene structure and conserved motif analysis

Conserved motifs in BcKNOX protein sequence were identified with the MEME tools with the parameters as described elsewhere (Li et al., 2022). The TBtools software was used to visualize structural information (Chen et al., 2020).

### Promoter sequence analysis

To analyze the cis-elements in the putative promoter regions, the 2,000-bp upstream sequences of the translation start site of *BcKNOX* genes were selected using the PlantCARE database (Lescot et al., 2002).

### Plant materials and treatments

Flowering Chinese cabbage cultivar ‘Youlv501’ was used to collected samples, which is planted in the greenhouse at South China Agriculture University. To analysis the expression pattern, roots, stem tip, leaves and flowers were collected. Seedlings with three true leaves were transplanted and sprayed with ZT (40mg/L), GA_3_ (200mg/L) or water (control), followed by sampling at 12h and flowering stage after treatment. All samples were immediately frozen in liquid nitrogen and stored at -80°C.

### RNA extraction, gene cloning and quantitative real-time PCR

The HiPure RNA Mini Kit (Magen, Guangzhou, China) was used to isolate total RNA following the manufacturer’s instructions. Primer pairs for quantitative real-time PCR (qRT-PCR) were designed using Primer3 (Untergasser et al., 2012) (**Supplementary Table S1**). First-strand cDNA was synthesized using the Hiscript QRT SuperMix (with gDNA Wiper; Vazyme, Nanjing, China). qRT-PCR was conducted on a LightCycler 480 real-time PCR system (Roche, Basel, Switzerland) using the SYBR ^®^ Green Premix Pro Taq HS qRT-PCR Kit (Accurate Biotechnology, AG11718, Hunan, China). The PCR protocol was described by (Ou et al., 2022). The comparative 2-ΔΔCT method was used to calculate relative expression values for each gene (Livak and Schmittgen, 2001).

### Subcellular localization analysis

The coding region lacking the stop codon of *BcKNOX1*, *BcKNOX3*, *BcKNOX4*, *BcKNOX5*, *BcKNOX7*, and *BcSTM* were fused to the N-terminus of the hGFP gene. The primers used for cloning are listed in **Supplementary Table S2**. The recombinant plasmids and localization signal (DsRed) were co-infiltrated into *Agrobacterium tumefaciens* strain GV3101 and then infiltrated *Nicotiana benthamiana* leaves (Sparkes et al., 2006). After two days, a laser scanning confocal microscopy (Axioimager.D2) was used to detect green fluorescent protein and DsRed protein.

### Correlation analysis of gene expression

The correlation analysis among transcription factors, GA metabolic genes, CTK metabolic genes, and some genes encoding cell wall structural proteins was conducted *via* Pearson’s correlation coefficients. To visualize the relationship, the R soft was used.

### Yeast two-hybrid assay

The coding sequences (CDS) of *BcRGA1* and *BcGRL1* were cloned in pGADT7 vector. Similarly, the *BcKNOX1* coding region was cloned into pGBKT7 vector. The primers used for cloning are listed in **Supplementary Table S3**. The vectors were co-transformed into yeast strain Y2HGold to generate fusion proteins.

### Bimolecular fluorescence complementation assay

The coding region of *BcRGA1*, *BcRGL1*, and *BcKNOX1* lacking stop codon were amplified by PCR. The primers used for cloning are listed in **Supplementary Table S4**. The *Agrobacterium* stain harboring the recombinant plasmid and DsRed were co-infiltrated into young *Nicotiana benthamiana* leaves (Ding et al., 2015). Three days after infiltration, a laser scanning confocal microscopy (Axioimager.D2) was used to detect green fluorescent protein and DsRed protein.

### Statistical analysis

Data was subjected to analysis of variance and the means were compared using Duncan’s test considering P<0.05 as the significance threshold using SPSS software (SPSS, Chicago, IL, USA). All results represented as the mean ± standard error of the mean of at least three replications.

## Results

### Genome-wide identification of flowering Chinese cabbage KNOX genes

**Figure 1:**
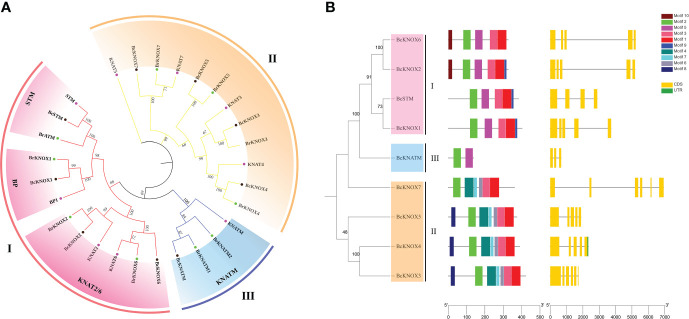
Phylogenetic analysis, Schematic representation of protein and gene structure. **(A)** Phylogenetic analysis of the KNOX families in *A thaliana*, *B rapa*, and *B campestris*. The tree was clustered into three subfamilies. **(B)** Schematic representation of protein and gene structure of *Brassica campestris* KNOX gene.

In Arabidopsis thaliana genome, nine *KNOX* gene were identified. With the nine AtKNOX proteins as queries, putative flowering Chinese cabbage *KNOX* genes were identified using BLASTP program based on conserved domain and named according their orthology with the reported isoform in Arabidopsis thaliana. The results of sequence analysis showed that the polypeptide lengths of the putative BcKNOXs ranged from 139 to 423 amino acids, isoeletric point (pI) values ranged from 4.68 to 6.28 and molecular weights ranged from 15.84 kDa to 46.62 kDa (**Table 1**). WoLF PSORT was used to analyze the subcellular location, which exhibited that all BcKNOXs were nuclear proteins (**Table 1**). Additionally, we selected BcKNOX1, BcKNOX3, BcKNOX4, BcKNOX5, BcKNOX7, and BcSTM to confirm the subcellar localization. As showed in **Figure S1**, the six proteins were nuclear proteins. The full-length sequences of the proteins were used to investigate the phylogenetic relationship of KNOX among *A. thaliana*, *B. rapa*, and *B. campestris* by constructing a rootless neighbor-joining phylogenetic tree. The tree contained 9 *Arabidopsis*, 9 *B. rapa*, and 9 *B. campestris* KNOX proteins (**Figure 1A**). The family was grouped into three subfamilies (**Figure 1A**). BcKNOX1, BcKNOX2, BcKNOX6, and BcSTM were ascribed to subfamily I. BcKNOX3, BcKNOX4, BcKNOX5, and BcKNOX7 were clustered into subfamily II. In addition, BcKNATM belonged to subfamily III (**Figure 1A**).

**Table 1:** Physicochemical properties of *KNOX* genes in flowering Chinese cabbage.

Gene	CDS (bp)	AA	pI	MW (KD)	Subcellular localization
***BcKNOX1***	1209	403	6.18	46.41	nucl: 14
***BcKNOX2***	984	328	4.91	36.93	nucl: 13, pero: 1
***BcKNOX3***	1269	423	5.81	46.62	nucl: 14
***BcKNOX4*** ***BcKNOX5*** ***BcKNOX6*** ***BcKNOX7*** ***BcSTM*** ***BcKNATM***	1170 1125 981 1086 1152 417	390 375 327 362 384 139	6.28 5.77 5.15 5.58 6.12 4.68	43.61 41.91 36.58 40.09 43.10 15.84	nucl: 14 nucl: 14 nucl: 14 nucl: 12, plas: 1, cysk: 1 nucl: 14 nucl: 6, cyto: 4, chlo: 2, extr: 2

### Conserved motifs gene structure of *BcKNOX* genes

The *BcKNOX* gene family differed in the number of introns and exons (**Figure 1B**). We used the NCBI-CDD database and Pfam database to analyze the conserved domain of BcKNOXs respectively. Multiple sequence alignment exhibited that the four conserved domains: KNOXI, KNOXII, ELK, and HD domain were present in all the BcKNOX proteins except for BcKNATM (**Figure S2**). BcKNOXs contained the KNOXI and KNOXII domains in their N-terminal region, while the C terminus possed ELK and HD domains. In addition, BcKNATM protein lacked ELK and HD domains. MEME program was used to investigated the conserved motifs. A total of 10 conserved motifs were predicted in BcKNOXs (**Figure 1B**). All BcKNOXs contained motifs 2; motif 1 and 3 were found in majority of BcKNOXs except for BcKNATM which belonged to subfamily III; motif 5 existed in members of subfamily I and III; motif 4, 6, and 7 were present in subfamily II. Furthermore, motif 8 only existed in BcKNOX3, BcKNOX4, and BcKNOX5; motif 9 was only present in BcKNOX1, BcKNOX2, and BcSTM; motif 10 only were found in BcKNOX2 and BcKNOX6.

### Chromosomal distribution and collinearity analysis of *BcKNOX* genes

**Figure 2:**
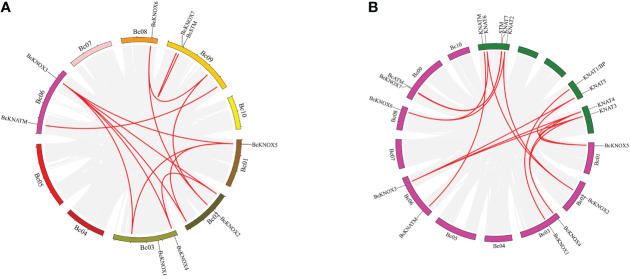
Collinearity analysis of among KNOX gene family members. **(A)** Distribution of segmentally duplicated *BcKNOX* genes on *B campestris* chromosomes. Gray lines indicate collinear blocks in the whole B. campestris genome and red lines indicate duplicated *BcKNOX* gene pairs. **(B)** Collinearity of *Arabidopsis thaliana* and *Brassica campestris*. Red lines indicate the syntenic gene pairs.

The chromosomal locations of the *BcKNOX* genes in *Brassica campestris* was mapped, and the physical mapping of *BcKNOXs* on 10 chromosomes revealed that an uneven distribution (**Figure S3**). The *BcKNOXs* were distributed on just 6 chromosomes. Chromosomes 3, 6, and 9 contained 2 genes. Only one gene was mapped onto chromosomes 1, 2, and 8, while no *KNOX* genes were located in the other chromosomes. To detect the evolution of the *BcKNOX* gene family in *B. campestris*, segmental and tandem duplication were analyzed. As shown in **Figure 2A**, more than ten pairs of *BcKNOX* genes were segmentally duplicated, for example, *BcKNOX3*/*BcKNOX4*, *BcKNOX3*/*BcKNOX5*, *BcKNOX2*/*BcKNOX6*. Chromosomes 4, 5, 7, and 10 did not exist any duplicated genes. Moreover, the results of synteny map showed that fifteen pairs of syntenic orthologous genes were matched between *A. thaliana* and *B. campestris* (**Figure 2B**).

### Analysis of cis-acting elements in the promoter regions of *BcKNOX* gene

**Figure 3:**
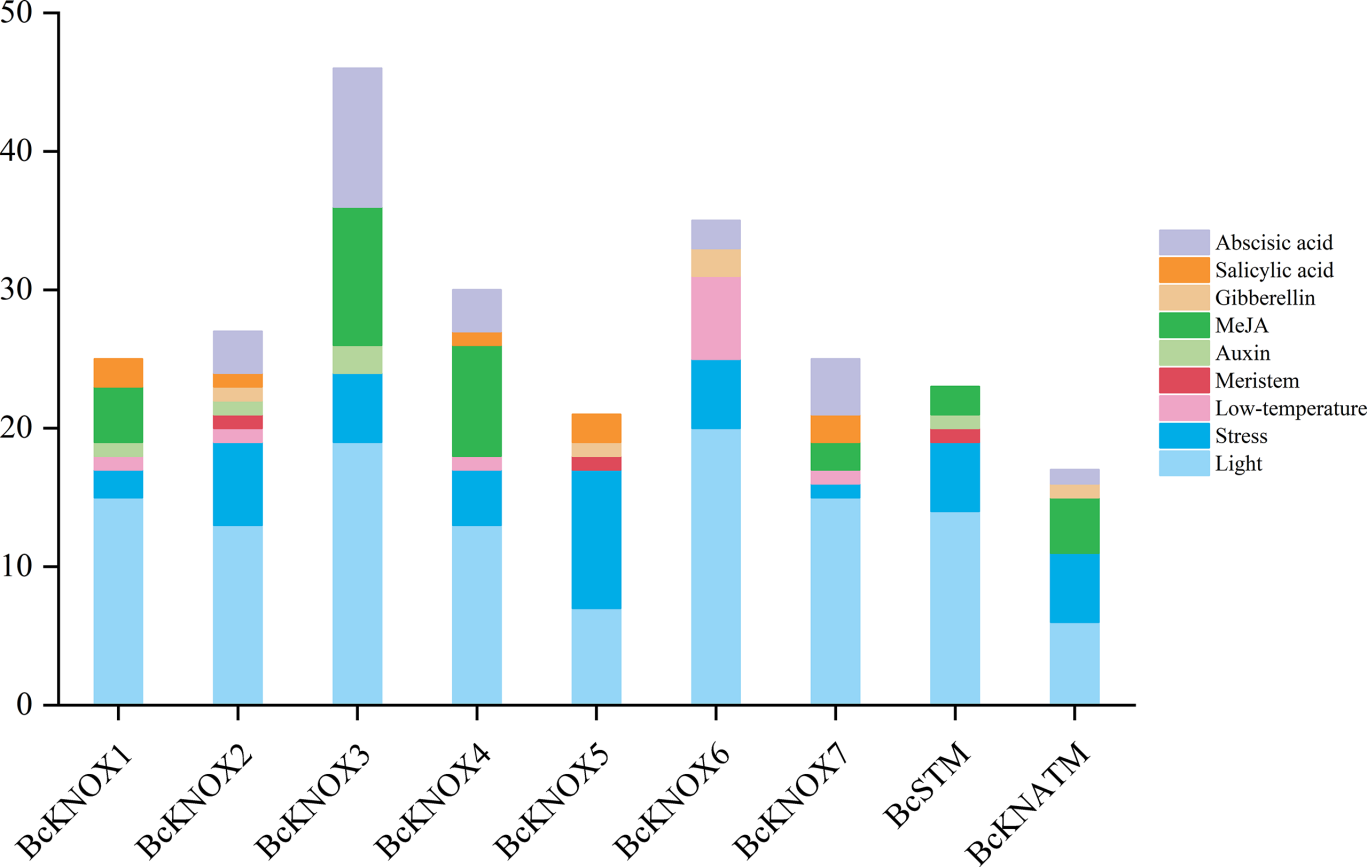
Analysis of cis-elements in the *BcKNOX* promoters.

The cis-regulatory elements in promoter regions are closely associated with responses to environmental factors and phytohormones. To explore the regulatory mechanisms and potential roles of BcKNOXs, the 2.0 Kb upstream sequences from the transcription start site were downloaded and detected using the Plant CARE database (Lescot et al., 2002). As show in the **Figure 3**, stress-related and light elements were found in the promoters of all *BcKNOX* genes. Low-temperature-related elements located in the promoters of *BcKNOX1*, *BcKNOX2*, *BcKNOX4*, *BcKNOX6*, and *BcKNOX7*. *BcKNOX2*, *BcKNOX5*, and *BcSTM* contained meristem-related cis-elements. Furthermore, several cis-elements were active in response to hormone, including ABA, SA, GA, methyl jasmonate, and auxin. Among these hormone-related cis-elements, the GA-responsive elements were existed in *BcKNOX2*, *BcKNOX5*, *BcKNOX6*, and *BcKNATM*; the auxin-responsive elements were present in *BcKNOX1*, *BcKNOX2*, *BcKNOX3*, and *BcATM*.

### Expression patterns of the *BcKNOX* genes during flowering Chinese cabbage development

**Figure 4:**
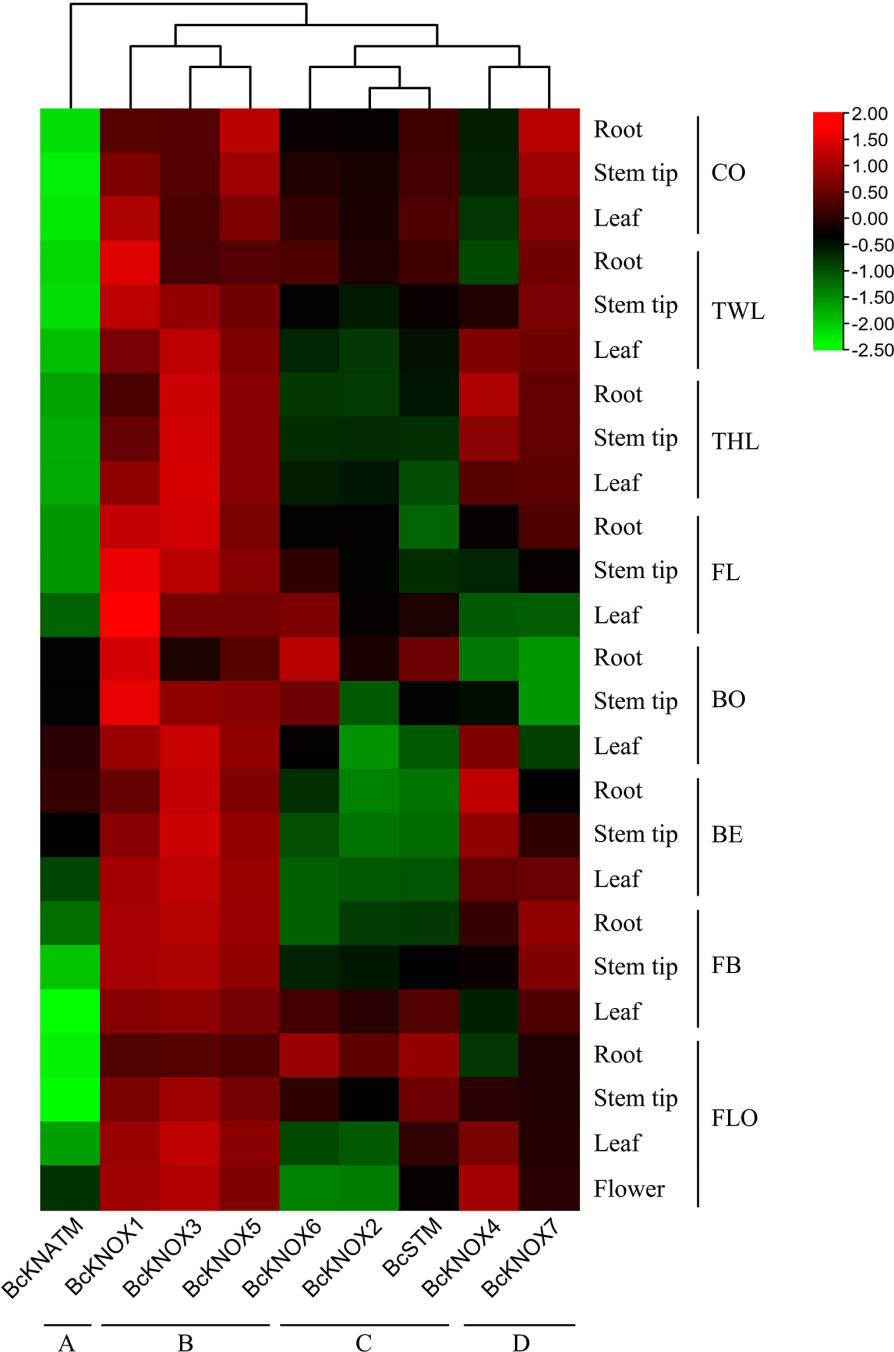
Heatmap of *BcKNOX* genes during flowering Chinese cabbage development. The *BcKNOX*s were divided into four groups. CO, cotyledon stage; TWL, two-leaf stage; THL, three-leaf stage; FL, four-leaf stage; BO, bolting stage; BE, bud emergence stage; FB, fast bolting stage; FLO, flowering stage.

To preliminarily analyze the functional roles of *BcKNOXs* in stalk development of flowering Chinese cabbage, the expression profiles of *BcKNOX* genes were investigated in different tissue at several stages. A heatmap was generated to visualize the expression patterns of individual *BcKNOX* genes according to the qRT-PCR data. The heatmap displayed that these *BcKNOX* genes could be divided into four groups according to their expression profiles (**Figure 4**). Group A contained *BcKNATM*, which showed low transcription level among the tested tissues at seven stages. *BcKNOX1*, *BcKNOX3*, and *BcKNOX5* belonged to Group B and they exhibited high expression levels in four tested tissues during the whole development stage. Group C had *BcKNOX2*, *BcKNOX6*, and *BcSTM*, which belonged to subfamily I. Their expression profiles showed that they had high transcript levels in roots at bolting and flowering stage, especially *BcKNOX6* and *BcSTM*, suggesting that these genes had strong preferential expression. Group D included *BcKNOX4* and *BcKNOX7*. Among the two genes, *BcKNOX4* showed high expression level in root, leaf, and stem tip during three-leaf, bud emergence, and flowering stage. In addition, during cotyledon, two-leaf, three-leaf, and fast bolting stages, *BcKNOX7* exhibited high expression level, while showed low transcript level at bolting stage.

### Expression of *BcKNOX* genes in response to cold stress

**Figure 5:**
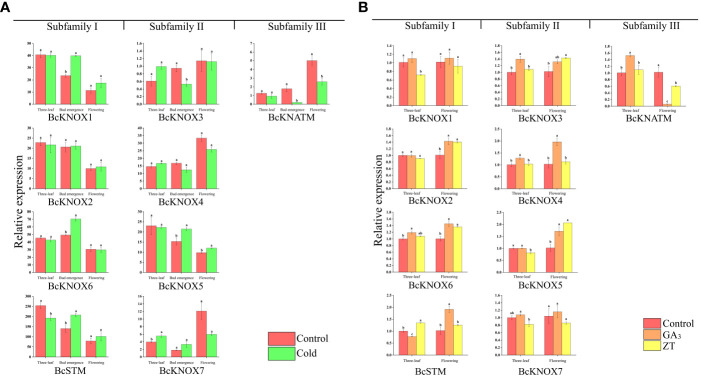
The expression patterns of BcKNOX genes were analyzed under cold, ZT and GA_3_ treatment. **(A)** Transcript levels of *BcKNOX*s in response to cold stress at three-leaf stage, bud emergence and flowering stage. **(B)** *BcKNOX* transcript levels of stem tip in response to ZT and GA_3_. Samples were collected at three-leaf and flowering stage after exogenous hormone treatment, with water used as a control. The lowercase letters indicate significance at p < 0.05.

Low temperature is an important environmental factor for stalk elongation and flowering in many winter annuals and biennials (Hébrard et al., 2013; Mathieu et al., 2014; Liang et al., 2018). Meanwhile, our previous studies have shown that cold treatment promoted bolting and flowering of flowering Chinese cabbage by inducing the GA levels in the shoot apical meristems (Song et al., 2019; Wang et al., 2022). To determine whether cold stress have effect on *BcKNOX* gene transcription, we investigated and compared the expression profiles of *BcKNOX* genes in response to cold tress among three-leaf, bud emergence, and flowering stages (Huang et al., 2017). As shown in **Figure 5A**, among the three stages, these *BcKNOX* genes exhibited different patterns upon cold treatment. It was worth noting that cold treatment displayed no significant effect on the transcript levels of *BcKNOX2* and *BcKNOX4* compared with control at three stages. The transcription level of *BcSTM* was considerably repressed at three-leaf stage. Moreover, *BcKNOX1 BcKNOX5*, *BcKNOX6*, and *BcSTM* were significantly upregulated at bud emergence stage. The transcription of *BcKNOX5* was also induced by cold treatment at flowering stage. Meanwhile, *BcKNOX3* and *BcKNATM* were significantly repressed at bud emergence stage. At three-leaf stage, cold treatment induced the expression of *BcKNOX7*, while *BcKNATM* was suppressed at flowering stage.

### Effect of phytohormone treatment on *BcKNOX*’s expression During the bud differentiation and flowering period

To investigate the potential effect of plant hormones on the transcript abundance of *BcKNOXs* at the bud differentiation and flowering stage, we estimated the transcript level after treatment with ZT and GA_3_ using RT-qPCR. As shown in **Figure 5B**, *BcKNOXs* showed various expression patterns over time in response to ZT and GA_3_. At three-leaf stage, ZT treatment significantly decreased the transcript level of *BcKNOX1*. Furthermore, the transcript abundance of *BcKNOX2* and *BcKNOX5* were downregulated in response to ZT at three-leaf stage, whereas treatment with GA_3_ and ZT promoted the expression level of *BcKNOX2* and *BcKNOX5* at flowering stage. No matter at three-leaf or flowering stage, the transcription of *BcKNOX4* was strongly induced by GA_3_ treatment. GA_3_ considerably induced the expression of *BcKNOX6* at three-leaf and flowering stage. Furthermore, *BcKNOX6* was induced in response to ZT treatment at flowering stage. *BcKNOX3* was remarkably upregulated by GA_3_ at three-leaf stage and was also promoted in response to ZT at flowering stage. The transcript abundance of *BcSTM* remained at lower levels compared with control in response to GA_3_ treatment at three-leaf stage, while ZT significantly increased the expression level of *BcSTM*. At flowering stage, GA_3_ treatment also induced the expression of *BcSTM*. *BcKNOX7* expression showed no significant difference under ZT and GA_3_ treatment. With regard to *BcKNATM*, GA_3_ considerably increased its expression level at three-leaf stage, but *BcKNATM* was suppressed by GA_3_ and ZT treatment at flowering stage (**Figure 5B**). In leaf, all of *BcKNOX* genes exhibited lower transcript levels in response to ZT treatment compared with that of control at three-leaf stage (**Figure S4**). The transcription of *BcKNOX2*, *BcKNOX5*, *BcKNOX6*, *BcKNOX7*, and *BcSTM* were also repressed by GA_3_ treatment at the same stage. In addition, at flowering stage, ZT decreased the expression levels of *BcKNOX1*, *BcKNOX2*, *BcKNOX3*, *BcKNOX4*, *BcKNOX5*, and *BcKNOX7*, whereas *BcSTM* and *BcKNATM* transcription were promoted (**Figure S4**).

### Correlation analysis of *BcKNOX* genes

**Figure 6:**
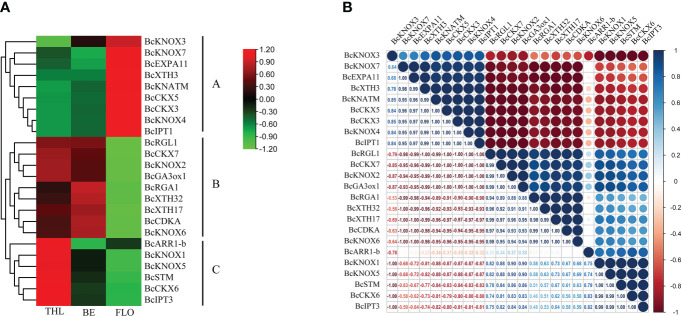
Expression patterns analysis and correlation of KNOX genes in flowering Chinese cabbage. **(A)** Heatmap of RNA-seq transcript abundance patterns of *BcKNOX*s and bolting related genes at three developmental stages. THL, three leaf stage; BE, bud emergence; FLO, flowering stage. **(B)** Correlative analysis of *BcKNOX* genes and bolting-related genes in three representative stages. R > 0.5 indicates a positive correlation; R < -0.5 indicates a negative correlation.

The stalk of flowering Chinese cabbage is the main product organ, whose development consists of bolting and flowering. Both of them are closely associated with the product quality and yield. Therefore, for understanding the possible correlation of the *BcKNOX*s and bolting-related genes, we investigated the transcription level of the nine *BcKNOXs* and the bolting-related genes. In addition, a comprehensive correlation analysis of these genes was performed. As shown in **Figure 6A**, nine *BcKNOXs* showed preferential expression at three-leaf, bud emergence, and flowering stages, which was consistent with the expression pattern of the bolting-related genes, exhibiting that these *BcKNOX*s had relevance to bolting in flowering Chinese cabbage. According to the correlative expression patterns, there were three different clusters (clusters A-C). The genes of gibberellins synthetase (*BcGA3ox1*), negative regulator of GA signaling (*BcRGA1* and *BcRGL1*), cytokinin oxidase (*BcCKX7*), cyclin-related gene (*BcCDKA*), and xyloglucan endotransglycosidase/hydrolase (*XTH17* and *XTH32*) clustered in cluster B and were highly expressed at three-leaf and bud emergence stages compatible with the expression pattern of *BcKNOX2* and *BcKNOX6* (**Figure 6A**). Moreover, *BcKNOX2* and *BcKNOX6* were strongly associated with these genes. Remarkably, *BcKNOX1*, *BcKNOX5*, and *BcSTM*, which clustered with cytokinin biosynthesis and metabolic genes (*BcIPT1* and *BcCKX6*) and cytokinin response regulator gene (*BcARR1-b*) (cluster C) were highly expressed at three-leaf stage. As shown in **Figure 6B**, *BcKNOX1* and *BcKNOX5* also showed high correlation level with gibberellins synthetase (*BcGA3ox1*). Besides, *BcKNOX1*, *BcKNOX2*, and *BcKNOX5* showed high correlation coefficients within the CTK metabolic genes (*BcCKX6* and *BcIPT3*). Furthermore, *BcKNOX3*, *BcKNOX4*, *BcKNOX7*, and *BcKNATM* were divided in cluster A, and highly expressed at flowering stage (**Figure 6A**). In addition, these *BcKNOXs* had high correlation with structural genes (*EXPA11* and *XTH3*) and CTK metabolic genes (*BcIPT1*, *BcCKX3*, and *BcCKX5*).

### *BcKNOX1* interacts with *BcRGA1* and *BcRGL1*

**Figure 7:**
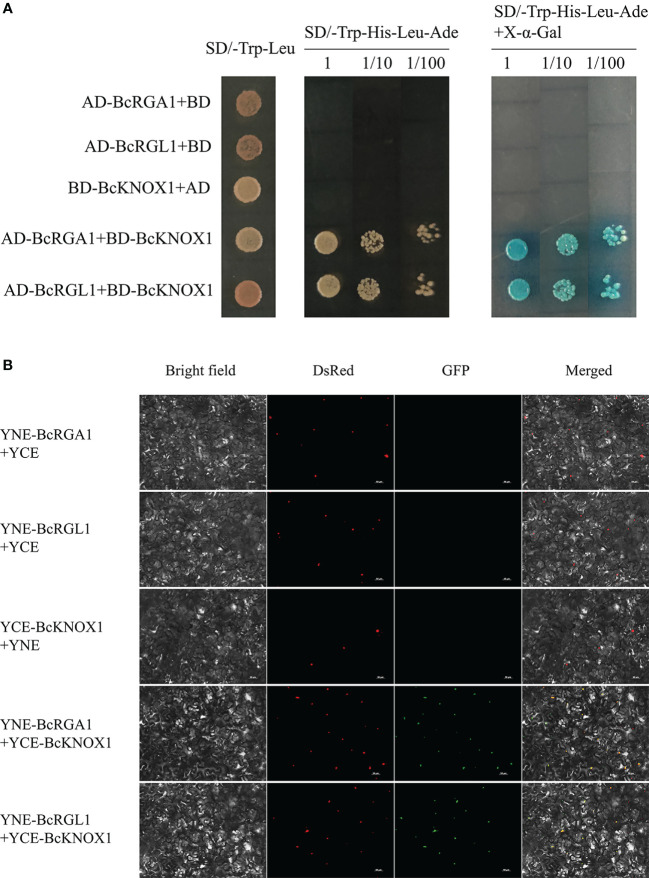
Yeast two-hybrid and biomolecular fluorescence complementation assays to determine protein interaction. **(A)** Interactions between BcKNOX1 and BcRGA1 and BcRGL1 in yeast cells. *BcKNOX1* coding region was fused in frame to the coding sequence of the GAL4 DNA binding domain (BD), and coding sequences of *BcRGA1* and *BcRGL1* were fused to the coding sequence of GAL4 activation domain (AD). The numbers above the images represent cell densities obtain by 0, 10-, and 100-times dilutions. Positive bacteria were stained using X-α-Gal. **(B)** Bimolecular fluorescence complementary assay was used to detect the interactions of BcKNOX1 (fused with C-terminal fragment of GFP) with BcRGA1 and BcRGL1 (fused with N-terminal fragment of GFP). The nuclei were stained with DsRed. Bar = 50μm.

Gibberellins are important orchestrator of plant growth and development. DELLAs are negative regulators of GA signaling, which function as coactivators or repressors by interacting with other transcription factors (Levesque et al., 2006; Cui et al., 2007; Fukazawa et al., 2014). Previous study have showed that BP/KNAT1 physically interacts with DELLA (Felipo-Benavent et al., 2018). To determine the protein interactions among BcKNOX1 and DELLAs in flowering Chinese cabbage, we conducted Y2H experiments. In yeast, BcKNOX1 interacted with BcRGA1 and BcRGL1 as shown in **Figure 7A**. To further verify the Y2H result *in vivo*, a biomolecular fluorescence complementation assay was performed. As shown in **Figure 7B**, the green fluorescent protein (GFP) fluorescence located in the nuclei of plant cells which confirmed that the interactions among BcKNOX1, BcRGA1 and BcRGL1.

## Discussion

The *KNOX* gene was first identified in maize and has since been characterized in many species (Vollbrecht et al., 1991; Hay and Tsiantis, 2010; Frangedakis et al., 2017). In this study, 9 *BcKNOXs* were obtained in flowering Chinese cabbage genome which was consistent with the number of *KNOX* genes in Arabidopsis thaliana. While, 13 Rice *KNOX* genes, 15 Poplar *KNOX* genes, and 22 Apple *KNOX* genes had been characterized up to date (Jain et al., 2008; Jia et al., 2020). The number of species harboring *KNOX* family in *Rice*, *Poplar*, and *Apple* was higher than the number in *Arabidopsis* and *Brassica campestris*, which may result from gene duplication. This suggests that segmental and tandem duplications are also known to contribute mainly in duplication modes during gene family expansion (Xu et al., 2020). In flowering Chinese cabbage genome, more than ten pairs of *BcKNOX* genes were segmentally duplicated. Chromosomes 4, 5, 7, and 10 did not exist any duplicated genes. For the evolution of plants, gene duplication is not only the motivation, but it has a prominent role in the process of genomic rearrangements (Vision et al., 2000). The *KNOX* genes in *Arabidopsis* are divided into three classes, class I, class II, and class M (Mukherjee et al., 2009). A rootless neighbor-joining phylogenetic tree was constructed using the full-length sequences of the KNOX proteins form *Arabidopsis*, *B. rapa*, and *B. campestris*. *BcKNOX*s homologous can also be clustered into three subfamilies based on sequences and gene structure. In addition, the majority of BcKNOXs except for BcKNATM contained four conserved domains (**Figure S2**). That implied *KNOX* is an ancient gene family and conserved highly.

The abundances of *BcKNOXs* differed at various developmental stages and *BcKNOXs* displayed different expression profiles suggesting that they have distinct roles in different tissues during different stages. In flowering Chinese cabbage, bolting is the most obvious characteristic. Previous studies had showed that stalk development can be classified into five stages based on bolting (Huang et al., 2017; Song et al., 2019). In this study, we primarily focused on three representative periods, three-leaf, bud emergence, and flowering stage. Among them, three-leaf and bud emergence stages were the key stage for bud differentiation and stalk development respectively (Huang et al., 2017; Guan et al., 2021). The results showed that *BcKNOX1*, *BcKNOX3*, and *BcKNOX5* were highly expressed in all tested tissues at each stage. Furthermore, *BcKNOX2*, *BcKNOX6*, and *BcSTM* had comparatively high expression levels in root of bolting and flowering stage, whereas *BcKNOX4* not only showed high abundance level at three-leaf and bud emergence stage but also in leaf and flower tissues of flowering stage. In addition, correlation analysis showed that *BcKNOX1*, *BcKNOX2*, *BcKNOX5*, and *BcKNOX6* had comparatively high correlation with gibberellins synthetase gene (*GA3ox1*), and some cytokinin metabolic genes. Expansin protein (*ExPA*) and xyloglucan endotransferases (*XTH*) are essential factors in cell elongation (Wang et al., 2022). In previous study, *BcExPA11* and *BcXTH3* showed a significant upregulation by low temperature and GA_3_ (Huang et al., 2017; Guan et al., 2021; Wang et al., 2022). *BcKNOX3*, *BcKNOX4*, and *BcKNOX7* showed high correlation coefficients within *BcExPA11* and *BcXTH3*. Meanwhile, the targets of *KNOX* showed a statistically significant enrichment of categories involved in cell wall metabolism and response to hormones (Felipo-Benavent et al., 2018). KNOX proteins can bind targets through a cis-regulatory elements containing a TGAC core (TGACTGAC or TGACAGG/CT) (Bolduc et al., 2012; Zhao et al., 2020). We conducted cis-regulatory element analysis which showed that more than 5 cis-regulatory elements containing TAGC motif were predicted within the promoters of *BcEXPA11*, *BcXTH3*, *BcXTH32*, *BcGA3ox1*, *BcIPT1*, *BcIPT3*, and *BcCKX6* (**Table 2**). We speculate that BcKNOXs may regulate the expression of these gene to involve in stalk development of flowering Chinese cabbage.

**Table 2:** The number of cis-regulatory elements containing ATGC of important genes in flowering Chinese cabbage.

Gene	Number of cis-regulatory elements containing ATGC
***BcEXPA11***	9
***BcXTH3***	9
***BcXTH17***	3
***BcXTH32***	9
***BcGA3ox1***	6
***BcIPT1***	6
***BcIPT3***	5
***BcCKX3***	3
***BcCKX5***	3
***BcCKX6***	16
***BcCKX7***	4
***BcARR1-b***	3
***BcRGA1***	4
***BcRGL1***	6

*KNOX* genes affected the metabolic and signaling transduction pathway of CTK and GA, while their expressions were also regulated by CTK (Truernit and Haseloff, 2007). At three-leaf stage, *BcKNOX1* and *BcKNOX5* were significantly suppressed upon ZT treatment, whereas *BcSTM* was remarkably induced by ZT. The transcript abundances of BcKNOX2, *BcKNOX3*, *BcKNOX5*, and *BcKNOX6* were upregulated by ZT treatment at flowering stage. At three-leaf stage, the transcription level of *BcSTM* was increased in response to ZT. Several cis-elements associated with responses to low-temperature and gibberellin were predicated within the *BcKNOX*s promoters (**Figure 3**). Among them, *BcKNOX1*, *BcKNOX4*, *BcKNOX6*, and *BcKNOX7* shared low-temperature-responsive elements. Gibberellin-responsive elements located in *BcKNOX2*, *BcKNOX5*, *BcKNOX6*, and *BcKNATM*. Additionally, we investigated the expression patterns under cold and GA_3_ treatment. Low temperature is a vital environmental factor for stalk elongation and flowering in many winter annuals and biennials (Hébrard et al., 2013; Mathieu et al., 2014; Liang et al., 2018). The cold treatment promotes bolting and flowering by accelerating flower and bud emergence in flowering Chinese cabbage (Huang et al., 2017; Song et al., 2019). The expression level of *BcSTM* were downregulated in stem tip at three-leaf stage in response to cold treatment. At bud emergence stage, *BcKNOX* subfamily I members except for *BcKNOX2* and subfamily II member *BcKNOX5* were induced upon cold treatment. Besides, *BcKNOX4*, *BcKNOX7*, and *BcKNATM* were repressed by cold treatment at flowering stage. It has also been revealed that GA shows a significant impact on plant bolting and flowering. Exogenous GA_3_ treatment promotes stem elongation and bolting in brassica napus (Dahanayake and Galwey, 1999). Furthermore, GA_3_ treatment accelerates bolting and flowering of flowering Chinese cabbage (Song et al., 2019; Wang et al., 2022). *BcKNOX2*, *BcKNOX5*, and *BcKNOX6* were significantly upregulated in response to GA_3_ at flowering stage. No matter three-leaf or flowering stage, the transcription of *BcKNOX4* was strongly promoted by GA_3_. At three-leaf stage, the expression level of *BcSTM* was decreased under GA_3_ treatment. Furthermore, treatment with GA_3_ also induced *BcKNATM* expression at preceding stage, however, this gene was inhibited at flowering stage. Whereas, a previous study have shown that gibberellin did not affect *KNAT* expression (Truernit et al., 2006). The transcription regulation of *BcKNOX*s need to be further analyzed.

In Arabidopsis, DELLA proteins function as negative transcriptional regulators of GA signaling transduction by directly interacting with specific transcription factors (Locascio et al., 2013; La Rosa et al., 2014). In this study, *BcKNOX1* showed high correlation coefficients within BcRGA1 and BcRGL1 which play a vital role in the bud differentiation and bolting (Guan et al., 2021). Moreover, *BcKNOX1* could interact with *BcRGA1* and *BcRGL1* (**Figure 7**) similar to a previous study (Felipo-Benavent et al., 2018). DELLA interaction with transcription factors has been shown to either impair their ability or to promote targets transaction (de Lucas et al., 2008; Gallego-Bartolomé et al., 2012; Fukazawa et al., 2014; Marin-de la Rosa et al., 2015). Meanwhile, DELLA proteins had been reported to be recruited by type-B ARRs that act as positive regulators of cytokinin signal transduction (Marin-de la Rosa et al., 2015). Given *KNOX* and CTK play a vital role in regulating meristematic activity. We hypothesized that BcKNOX1 would compete with type-B ARRs for binding to DELLA and control different targets depending on whether DELLA proteins were present. Taken together, through systematic analyses, we speculated that *BcKNOX1*, *BcKNOX3*, *BcKNOX5*, and *BcSTM* played important roles in stalk development.

## Conclusion

In summary, we obtained 9 *KNOX* genes in flowering Chinese cabbage genome. A phylogenetic analysis exhibited that the *BcKNOX*s are divided into three subfamilies. Four conserved domains were widely present in the proteins. The subcellular location assay further confirmed that the majority of BcKNOXs were nuclear proteins. Three key genes (*BcKNOX1*, *BcKNOX3*, and *BcKNOX5*) showed high transcript abundance on tested tissues at various stages. The major part of *BcKNOX* genes displayed preferential expression patterns in response to low-temperature, ZT, and GA_3_ treatment, implying that they were involved in bud differentiation and bolting. *BcKONX1* had a high correlation with *BcRGA1* and *BcRGL1*. *BcKNOX1* and *BcKNOX5* also showed high correlation level with gibberellin synthetase (*BcGA3ox1*). Besides, *BcKNOX1*, *BcKNOX2*, and *BcKNOX5* showed high correlation coefficients with CTK metabolic genes (*BcCKX6* and *BcIPT3*). *BcKNOX4* also showed high correlation coefficients within *BcExPA11* and *BcXTH3*. In addition, BcKNOX1 interacted with BcRGA1 and BcRGL1, as confirmed by Y2H and biomolecular fluorescence complementation assay. This study has provided valuable information for the future functional roles analysis of flowering Chinese cabbage KNOX genes.

## Data availability statement

The datasets presented in this study can be found in online repositories. The names of the repository/repositories and accession number(s) can be found in the article/**Supplementary Material**.

## Author contributions

XO performed the experiments and analyzed the data. JZ, ZX, JL, and BH collected the materials for the experiment and provided useful suggestions. YH, YudW and RC participated in the design of the study. XO and YH wrote the manuscript. YH, RC, SS, WS and YW assisted in revising the manuscript. All authors contributed to the article and approved the submitted version.

## Funding

This work was funded by the National Natural Science Foundation of China (32072656) and (31972481), Key-Area Research and Development Program of Guangdong Province, China (2020B0202010006), and the China Agriculture Research System of MOF and MARA.

## Acknowledgments

We show many thanks to the group members of protected horticulture in South China Agriculture University for providing the laboratory and every week group meeting.

## Conflict of interest

The authors declare that the research was conducted in the absence of any commercial or financial relationships that could be construed as a potential conflict of interest.

## Publisher’s note

All claims expressed in this article are solely those of the authors and do not necessarily represent those of their affiliated organizations, or those of the publisher, the editors and the reviewers. Any product that may be evaluated in this article, or claim that may be made by its manufacturer, is not guaranteed or endorsed by the publisher.
