# Supplementary material for: Genome-wide identification of the KNOTTED HOMEOBOX gene family and their involvement in stalk development in flowering Chinese cabbage
Source: Front Plant Sci. 2022 Nov 10;13:1019884. doi: 10.3389/fpls.2022.1019884 (PMC9686407; doi:10.3389/fpls.2022.1019884)
Supplement: Supplementary file 2 [file Presentation_1.ppt]

## Slide 1
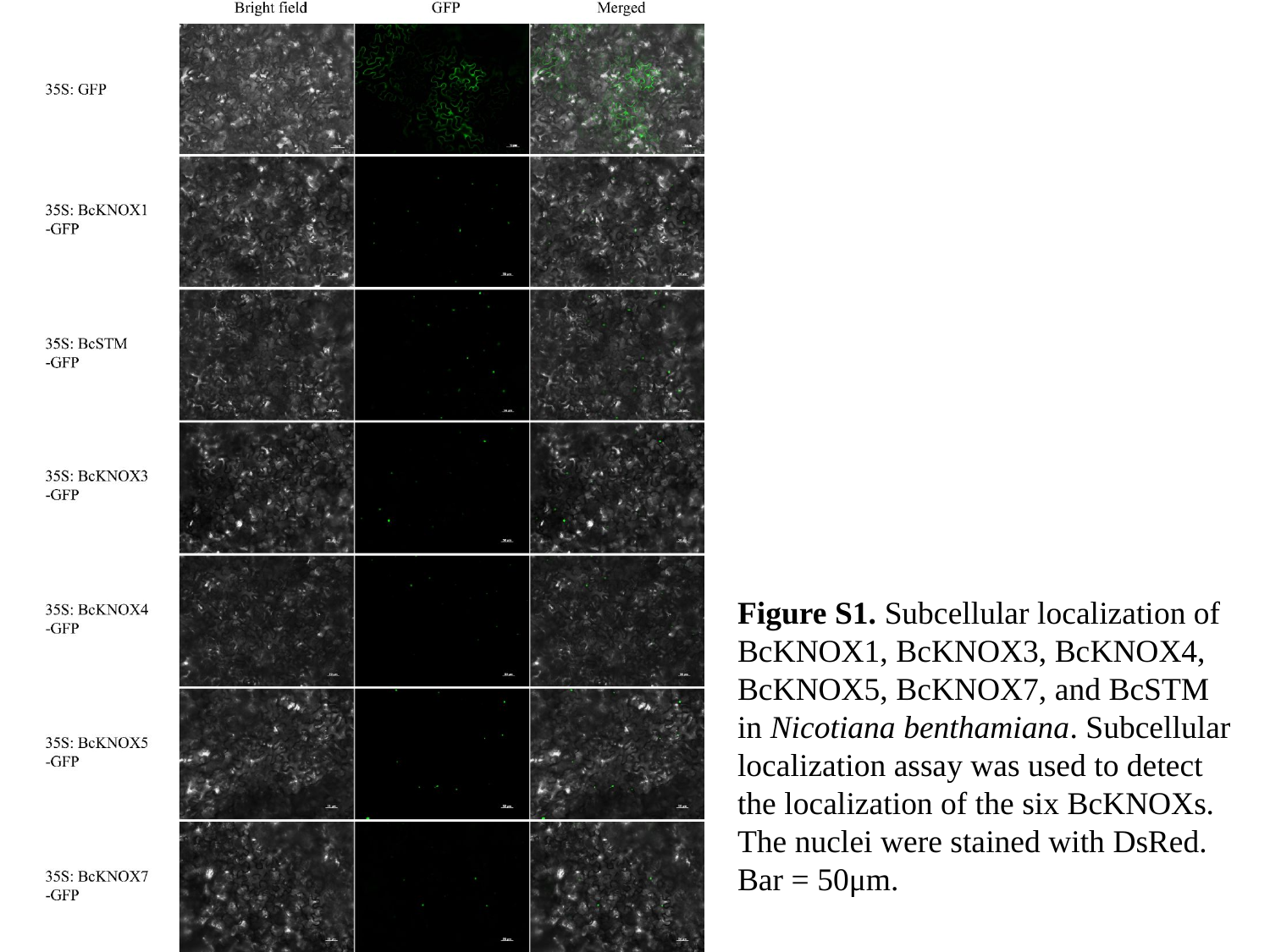

Figure S1. Subcellular localization of BcKNOX1, BcKNOX3, BcKNOX4, BcKNOX5, BcKNOX7, and BcSTM in Nicotiana benthamiana. Subcellular localization assay was used to detect the localization of the six BcKNOXs. The nuclei were stained with DsRed. Bar = 50μm.

## Slide 2
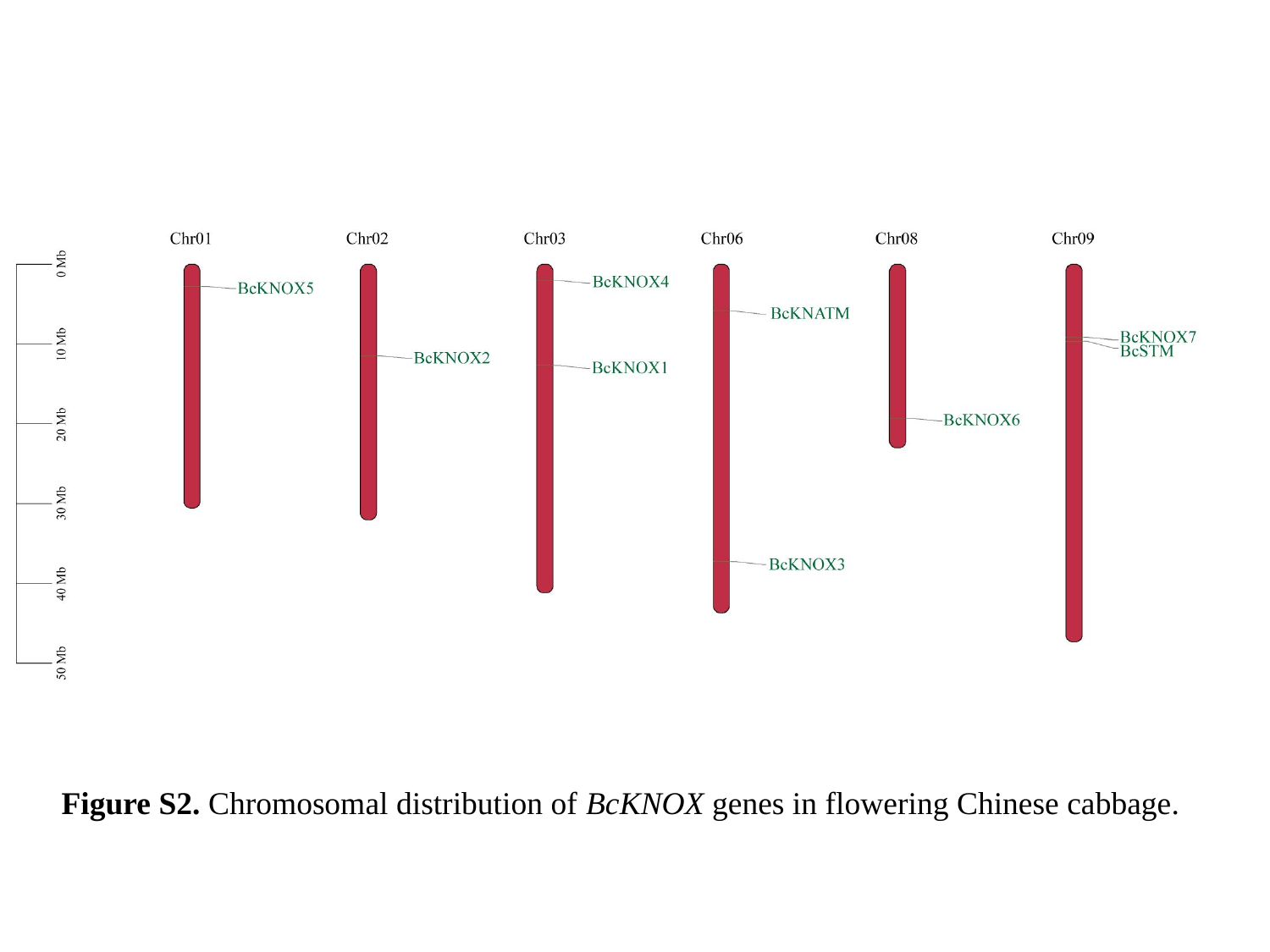

Figure S2. Chromosomal distribution of BcKNOX genes in flowering Chinese cabbage.

## Slide 3
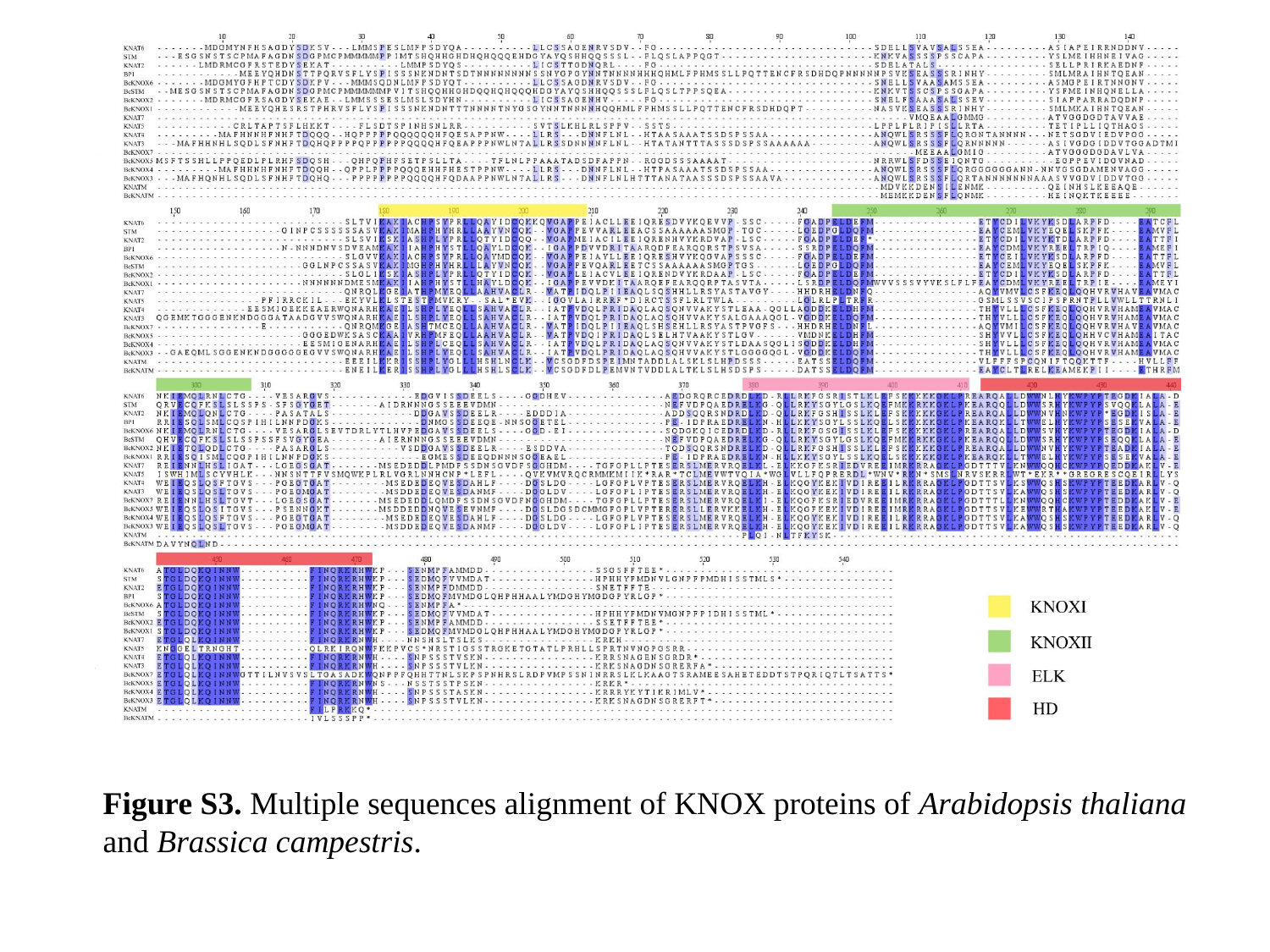

Figure S3. Multiple sequences alignment of KNOX proteins of Arabidopsis thaliana and Brassica campestris.

## Slide 4
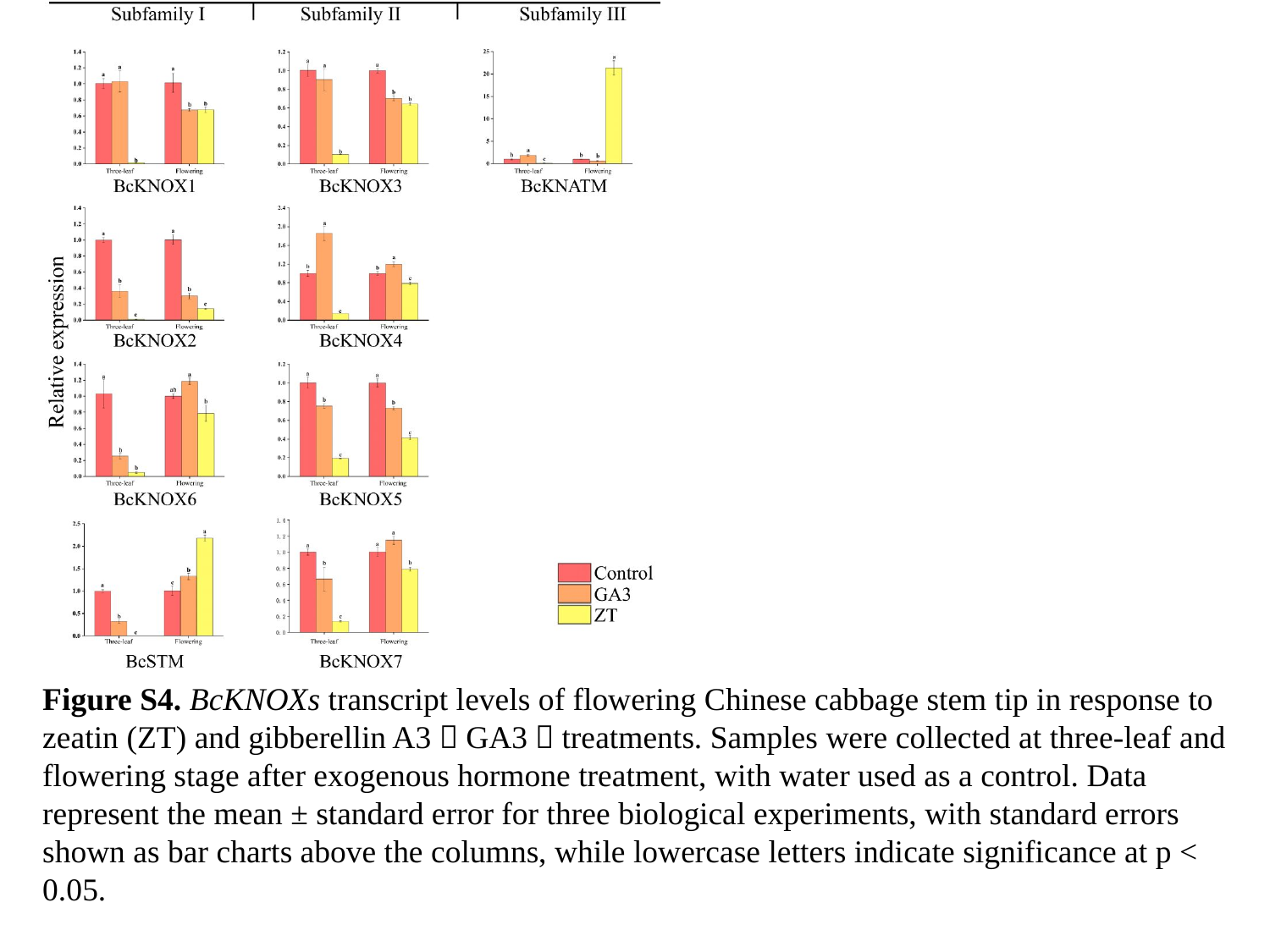

Figure S4. BcKNOXs transcript levels of flowering Chinese cabbage stem tip in response to zeatin (ZT) and gibberellin A3（GA3）treatments. Samples were collected at three-leaf and flowering stage after exogenous hormone treatment, with water used as a control. Data represent the mean ± standard error for three biological experiments, with standard errors shown as bar charts above the columns, while lowercase letters indicate significance at p < 0.05.
